# Surviving Ebola: A historical cohort study of Ebola mortality and survival in Sierra Leone 2014-2015

**DOI:** 10.1371/journal.pone.0209655

**Published:** 2018-12-27

**Authors:** Kevin Wing, Shefali Oza, Catherine Houlihan, Judith R. Glynn, Sharon Irvine, Clare E. Warrell, Andrew J. H. Simpson, Sabah Boufkhed, Alieu Sesay, Lahai Vandi, Sahr Charles Sebba, Pranav Shetty, Rachael Cummings, Francesco Checchi, Catherine R. McGowan

**Affiliations:** 1 Save the Children International, Kerry Town, Sierra Leone; 2 London School of Hygiene & Tropical Medicine, London, United Kingdom; 3 Division of Infection and Immunity, University College London, London, United Kingdom; 4 Rare and Imported Pathogens Laboratory, Public Health England, Porton, Wilts, United Kingdom; 5 Humanitarian Public Health Technical Unit, Save the Children, London, United Kingdom; University of Texas Medical Branch at Galveston, UNITED STATES

## Abstract

**Background:**

While a number of predictors for Ebola mortality have been identified, less is known about post-viral symptoms. The identification of acute-illness predictors for post-viral symptoms could allow the selection of patients for more active follow up in the future, and those in whom early interventions may be beneficial in the long term. Studying predictors of both mortality and post-viral symptoms within a single cohort of patients could also further our understanding of the pathophysiology of survivor sequelae.

**Methods/Principal findings:**

We performed a historical cohort study using data collected as part of routine clinical care from an Ebola Treatment Centre (ETC) in Kerry Town, Sierra Leone, in order to identify predictors of mortality and of post-viral symptoms. Variables included as potential predictors were sex, age, date of admission, first recorded viral load at the ETC and symptoms (recorded upon presentation at the ETC). Multivariable logistic regression was used to identify predictors. Of 263 Ebola-confirmed patients admitted between November 2014 and March 2015, 151 (57%) survived to ETC discharge. Viral load was the strongest predictor of mortality (adjusted OR comparing high with low viral load: 84.97, 95% CI 30.87–345.94). We did not find evidence that a high viral load predicted post-viral symptoms (ocular: 1.17, 95% CI 0.35–3.97; musculoskeletal: 1.07, 95% CI 0.28–4.08). Ocular post-viral symptoms were more common in females (2.31, 95% CI 0.98–5.43) and in those who had experienced hiccups during the acute phase (4.73, 95% CI 0.90–24.73).

**Conclusions/Significance:**

These findings may add epidemiological support to the hypothesis that post-viral symptoms have an immune-mediated aspect and may not only be a consequence of high viral load and disease severity.

## Introduction

The 2013–2015 Zaire Ebolavirus (EBOV) epidemic in West Africa infected more than 28 000 people, with over 50% of cases occurring within Sierra Leone[1]. While the case fatality from Ebola Virus Disease (EVD) has been estimated to be as high as 80% based on previous smaller outbreaks[2], the WHO reported an overall case fatality of around 65% in the West African outbreak[3]. Estimates of case fatality from individual Ebola treatment centres (ETC) ranged from 31% to 70%[3,4].

Descriptive analysis from treatment centres caring for patients during the West African outbreak has improved our understanding of common presenting features of EVD, which are now characterised into three stages; stage 1: non-specific symptoms, stage 2: gastrointestinal symptoms, and stage 3: neurological symptoms and organ failure[5]. Signs and symptoms associated with severe (advanced) EVD include: hiccups, confusion, depressed consciousness, seizures, difficulty breathing, and bleeding[6]. Follow-up of survivors of the West African epidemic, mostly from small cohorts, indicates a high frequency of debilitating post-viral symptoms[7–12], as well as considerable psychosocial challenges[13–16]. To date, however, there has been limited examination of which patient characteristics, and/or presenting symptoms or signs, are predictors of post-viral symptoms, and whether these are the same as predictors for mortality during the acute phase of the disease. The identification of acute-illness predictors for late-onset survival symptoms would allow the identification of patients for more active follow up, and those in whom early interventions may be beneficial in the long term, as well as furthering our understanding of the pathophysiology of survivor sequelae. Here, we analyse predictors of both mortality and sequelae within the same large cohort of patients cared for during acute illness and recovery in a single ETC.

Our primary aim was to identify risk factors for (i) mortality and (ii) ocular and musculoskeletal post-viral symptoms within a single EVD-infected cohort. Our secondary aim was to describe the types and prevalence of post-viral symptoms experienced by survivors, adding to emerging evidence on EVD sequelae.

## Methods

### Study design

This was an historical cohort study using de-identified data captured during routine clinical care of (i) people infected with EBOV and (ii) a subset of these who survived acute disease.

### Study participants and setting

The study population consisted of all people admitted to the 80-bed Kerry Town ETC in the Western Area Rural District, Sierra Leone, between 5 November 2014 and 31 March 2015. This ETC was operated by Save the Children International in partnership with the United Kingdom (UK) and Sierra Leonean governments, and the Cuban Medical Brigade.

The ETC admitted patients with suspected or previously lab-confirmed EBOV, mostly originating from the nearby Western Area Urban or Western Area Rural districts. Further details on admission, care and discharge procedures can be found in the Supporting Information and elsewhere[17,18]. All survivors (i.e. initially EBOV PCR-positive individuals who had three EVD symptom-free days and two consecutive EBOV PCR-negative tests and were discharged from the ETC) were invited to attend the survivor clinic which ran from 2 April 2015 until 30 June 2015, whether or not they had any symptoms (see Supporting information for full details).

**Fig 1:**
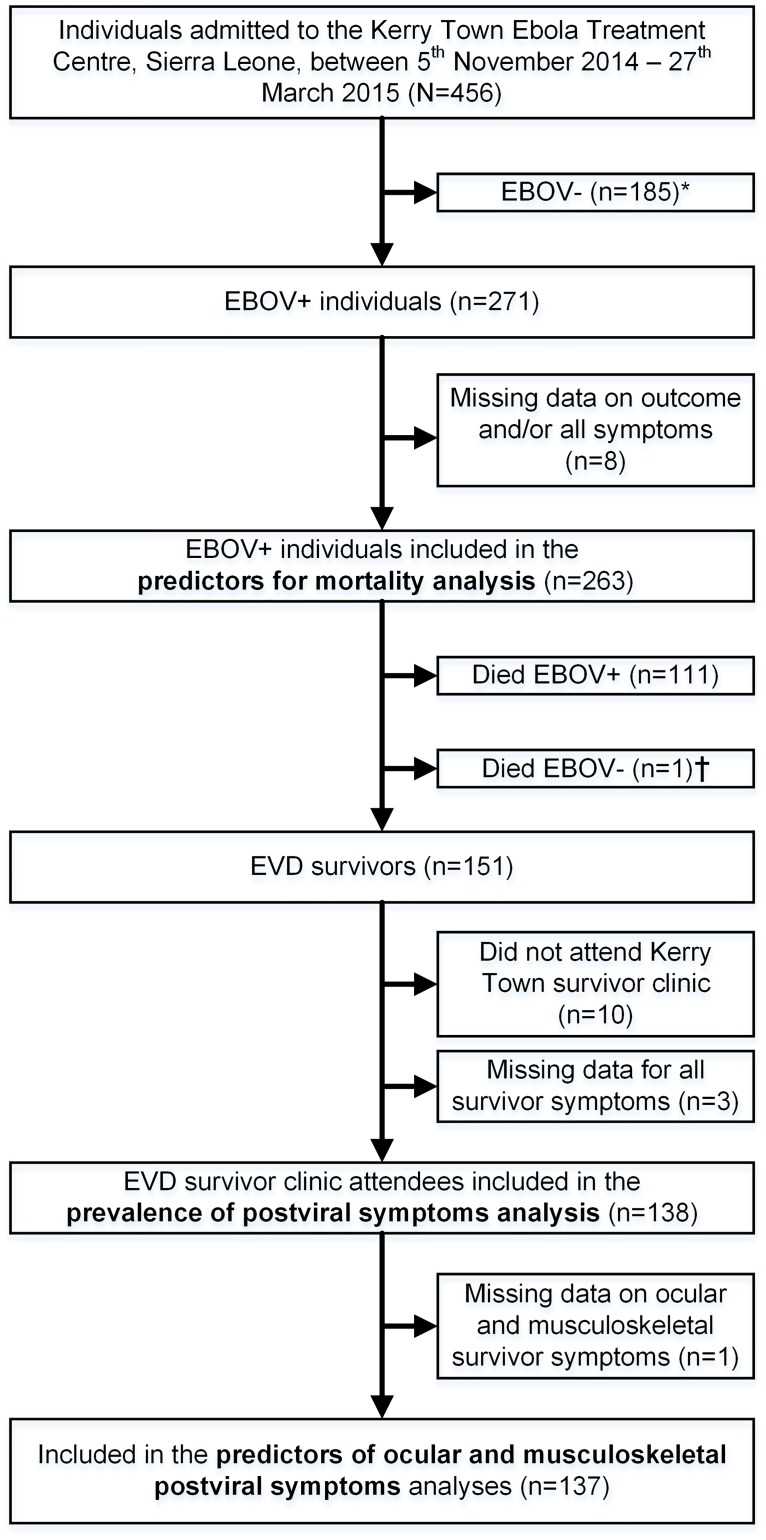
Flow diagram of individuals included in the analysis of EVD mortality and survival in Kerry Town, Sierra Leone, 2014–2015. *EBOV-: Admitted to the ETC as a suspect case based upon meeting EVD case definition. Laboratory test for EBOV performed at laboratory located at Kerry Town ETC and result was negative. Individual discharged (or referred) and not included in this study. **†Died EBOV-:** Person recovered from Ebola (i.e. three EVD symptom-free days and two consecutive EBOV-negative tests) but then died during the ETC discharge procedure. Classified as “recovered” in the mortality analysis, but not included as a survivor in the survivor analysis as did not survive to discharge from the ETC.

Patients who had a documented positive EBOV PCR test from the onsite Public Health England Laboratory and who were inpatients at the Kerry Town ETC were eligible for this study and were included in the analysis of risk factors for EVD mortality. Survivors among these were included in the post-viral symptoms analyses if they attended the survivor clinic at least once (Fig 1).

Subsets of the cohort of 263 participants included in this study have been included in previous publications: 150 in a study of survival in the ETC[19]; 112 survivors in a study of persistent viral excretion[20]; all 151 survivors in a study of long term mortality[21] and 123 survivors in a series of studies on household transmission[22–25] (See Supporting information for further details).

### Data collection

We used routinely-collected de-identified clinical data, originally recorded on standardised clinical record forms at the ETC, supplemented where applicable by case investigation form data carried by patients referred from other facilities. Diagnosis of EBOV infection was made using reverse-transcriptase-PCR (RT-PCR) assay performed using the Altona RealStar Filovirus RT-PCR Kit (Altona, Hamburg, Germany), after inactivation and manual RNA extraction, as previously described[19]. People who arrived at the ETC with case investigation forms from other facilities indicating they were EBOV PCR-positive were admitted to the ETC and retested by the onsite Public Health England laboratory.

At the survivor clinic, attendees were initially screened for acute infection as described previously[20]. Survivor clinic attendees were seen by a clinician who recorded the patients’ self-reported symptoms experienced in the preceding seven days on a standardised form and also recorded clinical signs, symptoms, and any clinical diagnoses made during the examination. A Krio translator was used if necessary. If requested by the attending clinician, blood samples were taken for haematological and biochemical analysis. Survivors with symptoms and/or a diagnosis requiring specialist input were referred to hospitals. A psychosocial assessment was also performed by a trained psychologist at the majority of survivor clinic visits. See Supporting Information for further data collection details.

### Ethics

The Sierra Leone Ethics and Scientific Review Committee granted ethical approval for this study. As this study used de-identified clinical data collected for the purposes of routine clinical care only, individual consent was not sought.

### Outcomes and risk factors

#### Mortality analysis

Variables included as potential risk factors were sex, age, date of admission, viral load on admission (EBOV RT-PCR cycle threshold value was used as a proxy) and any of the following symptoms or signs recorded upon admission: fever, fatigue/weakness, vomiting/nausea, diarrhoea, conjunctivitis/red eye, muscle/joint pain, headache, difficulty breathing, skin rash, hiccups, unexplained bleeding, or confusion. See S1 Text for further details on risk factors.

#### Post-viral symptoms analyses

For the EVD survivor post-viral symptoms analyses, we used only self-reported post-viral symptom data collected as part of routine clinical care at the survivor clinic, with symptoms selected from a standardised form following the question, “have you had any of the following symptoms within the previous seven days”.

For the risk factor analysis, we initially considered the three most prevalent types of post-viral symptoms reported in the literature as potential outcomes: ocular, musculoskeletal, and auditory [8–10]. Based on the results of our descriptive analysis of symptoms self-reported by our cohort we restricted this to any (self-reported) ocular symptom (which included any one of: ocular pain, photophobia, hyperlacrimation, loss of vision, foreign body sensation in the eye, red eye) and any (self-reported) musculoskeletal symptom (which included any one of: joint pain, back pain, muscle pain, movement problems, or jaw pain).

The potential risk factors included were sex, age, and information from the acute phase of infection: total days admitted to ETC, number of days between ETC discharge and survivor clinic attendance, viral load from the first test at the ETC, and having any one of the 10 symptom risk factors detailed in the mortality analysis section above upon presentation at the ETC during the acute-phase of infection.

### Statistical analysis

#### Analysis of risk factors for mortality and post-viral symptoms

Crude odds ratios (ORs) were calculated for the association between each specific outcome and each potential risk factor. An initial multivariable logistic regression model was then prepared that included the RT-PCR threshold value variable split into tertiles (included on an a-priori basis as key risk factor for all outcomes) and all variables listed in the “Mortality analysis” section above as potential risk factors[26]. For the mortality analysis, age and date of admission were included in the model as continuous variables. For the post-viral analyses, age, total days admitted to ETC, and days since discharge were included in the model as continuous variables. A final model was then obtained by removing all potential risk factors with p>0.2 from the fully-adjusted model in a backward-stepwise fashion (see S1 Text).

#### Missing data and sensitivity analyses

Missing risk factor data were assumed to be missing at random[27], and were accounted for using multiple imputation by chained equations (see S1 Text). We performed two RT-PCR sensitivity analyses: (i) one where RT-PCR value was not included a priori and (ii) one where RT-PCR was included as a continuous variable. We also repeated the analysis including acute-phase symptom risk factors recorded at any time during ETC stay (not just upon presentation), and using the lowest recorded RT-PCR value (equivalent to the highest viral load) during ETC stay (rather than the first recorded).

## Results

### Participants

Between 5 November 2014 and 27 March 2015, there were 456 total admissions to Kerry Town ETC, 271/456 (59%) of whom were EBOV PCR-positive. Of these, three had missing outcome data and five had missing data for all symptoms, leaving 263 people in the predictors for mortality analysis (Fig 1). 151/263 (57%) of these survived to ETC discharge, 141/151 (93%) attended the survivor clinic at least once, 137/141 (98%) of whom had sufficient symptom data for the post-viral symptoms analyses (Fig 1).

### Cohort description

The 263 eligible patients had a median age of 25 years (IQR 14–35) and 148/263 (56%) were female (Table 1). The most common symptoms recorded upon presentation at the ETC were fatigue/weakness (213 out of 242 people who had data available for the symptom, 88%) and fever (212/242, 88%). Vomiting/nausea (160/242, 66%), headache (149/242, 62%), muscle/joint pain (158/242, 65%), and diarrhoea (150/242, 62%) were also common (Table 1). Unexplained bleeding was less common (25/242, 10%), as was skin rash (10/242, 4%). Median length of stay at the ETC was nine days (IQR 6–14) for those who survived, compared with three days (IQR 2–5) for those who died.

**Table 1:** Predictors for mortality amongst all EBOV-positive people admitted to Kerry Town ETC.

		All n (%)	Recovered n (%)	Died	Crude OR* (95% CI^†^)	Multivariable^‡^ OR (95% CI)
**Total**^§^		263 (100)	152 (58)	111 (42)	-	-
**Gender**	Male	115 (44)	60 (52)	55 (48)	1	-
	Female	148 (56)	92 (62)	56 (38)	0.66 (0.41–1.09)	-
**Age in years**	<5	21 (8)	10 (48)	11 (52)	2.82 (1.19–7.00)	2.36 (0.48–11.64)
	5–14	48 (18)	35 (73)	13 (27)	0.95 (0.40–2.25)	0.92 (0.27–3.19)
	15–24	57 (22)	41 (72)	16 (28)	1	1
	25–34	71 (27)	34 (48)	37 (52)	2.79 (1.33–5.86)	3.80 (1.25–11.56)
	35–44	34 (13)	16 (47)	18 (53)	2.88 (1.19–7.00)	4.68 (1.27–12.28)
	45+	32 (12)	16 (50)	16 (50)	2.56 (1.04–6.32)	8.82 (2.17–35.87)
**Date of**	Nov 14	42 (16)	14 (33)	28 (67)	1	1
**Admission**	Dec 14	160 (61)	100 (63)	60 (38)	0.30 (0.15–0.61)	0.20 (0.06–0.64)
	Jan 15	34 (13)	22 (65)	12 (35)	0.27 (0.11–0.71)	0.18 (0.04–0.75)
	Feb/Mar 15	27 (10)	16 (59)	11 (41)	0.34 (0.13–0.93)	0.48 (0.10–2.24)
**RT-PCR cycle**	High	78 (36)	68 (87)	10 (13)	1	1
**threshold**^¶^ (n = 214)^#^	Med	68 (32)	44 (65)	24 (35)	3.71 (1.61–8.54)	5.90 (2.05–16.95)
	Low	68 (32)	10 (15)	58 (85)	32.85 (12.61–85.60)	84.97 (30.87–345.94)
Fever** (n = 242)	No	30 (12)	16 (53)	14 (47)	1	-
	Yes	212 (88)	125 (59)	87 (41)	0.83 (0.39–1.76)	-
**Fatigue/weakness** (n = 242)	No	29 (12)	18 (62)	11 (38)	1	-
	Yes	213 (88)	123 (58)	90 (42)	1.18 (0.54–2.58)	-
**Vomiting/nausea** (n = 242)	No	82 (34)	47 (57)	35 (43)	1	-
	Yes	160 (66)	94 (59)	66 (41)	0.93 (0.54–1.60)	-
**Diarrhoea** (n = 242)	No	92 (38)	57 (62)	35 (38)	1	-
	Yes	150 (62)	84 (56)	66 (44)	1.24 (0.72–2.16)	-
**Conjunctivitis/red eye** (n = 242)	No	145 (60)	90 (62)	55 (38)	1	-
	Yes	97 (40)	51 (53)	46 (47)	1.46 (0.86–2.49)	-
**Muscle/joint pain** (n = 242)	No	84 (35)	47 (56)	37 (44)	1	-
	Yes	158 (65)	94 (59)	64 (41)	0.86 (0.51–1.46)	-
**Headache** (n = 242)	No	93 (38)	47 (51)	46 (49)	1	1
	Yes	149 (62)	94 (63)	55 (37)	0.60 (0.35–1.02)	0.51 (0.23–1.15)
**Difficulty breathing** (n = 242)	No	197 (81)	113 (57)	84 (43)	1	-
	Yes	45 (19)	28 (62)	17 (38)	0.81 (0.42–1.57)	-
**Skin rash** (n = 242)	No	232 (96)	135 (58)	97 (42)	1	-
	Yes	10 (4)	6 (60)	4 (40)	0.93 (0.26–3.32)	-
**Hiccups** (n = 242)	No	203 (84)	120 (59)	83 (41)	1	-
	Yes	39 (16)	21 (54)	18 (46)	1.29 (0.66–2.53)	-
**Unexplained bleeding** (n = 242)	No	217 (90)	130 (60)	87 (40)	1	-
	Yes	25 (10)	11 (44)	14 (56)	1.94 (0.85–4.44)	-
**Confusion** (n = 242)	No	226 (93)	139 (62)	87 (38)	1	1
	Yes	16 (7)	2 (13)	14 (87)	11.29 (2.47–51.54)	15.93 (2.56–98.97)

*: Odds ratio. Multiple imputation (MI) used to account for missing data for all variables with missing data. MI model included all variables in this table and the outcome status.

^†^: Confidence interval

^‡^: An initial multivariable regression model was prepared that included all variables in this table. The final model presented here was obtained by removing variables from the initial fully-adjusted model in a backwards stepwise fashion, keeping only those variables with p≤0.2. Age was included as a continuous variable (multivariable-adjusted categorical results presented to aid interpretation of results).

^§^: Total = total number of EBOV-positive people admitted to Kerry Town ETC

^¶^: First recorded RT-PCR cycle threshold value at ETC (inverse indicator of viral load), categorised into tertiles of the distribution of the variable (Low: <18.6 cycles, medium: 18.6-<22.5 cycles, high: ≥22.5 cycles).

^#^: The figures in parentheses indicate the total number of individuals with any data recorded for that variable. Missing values for any variable with missing data were imputed using multiple imputation (see note 1). See S4 Table for a comparison of analysing the imputed data versus complete records only

**: All symptoms in first column of this table were recorded by clinical staff on presentation at the Ebola Treatment Centre.

### Mortality

#### Analysis of risk factors for mortality

RT-PCR cycle threshold (CT) was the strongest predictor of mortality (Table 1), with those with a low CT value (high EVD viral load) at ETC admission having 85 times higher odds of death than those with a high threshold value on admission, after multivariable adjustments. In addition, presenting at the ETC in a confused state was a strong predictor of mortality (OR 15.93, 95% CI 2.56–98.97). Mortality increased with age in adults, and was lower in those admitted in December and January than those admitted in November.

In a post hoc analysis we found that none of the 18 people with a CT value less than 15.9 upon admission survived, while none of the 29 people with a CT value greater than 29.0 died.

### Survivors’ post-viral symptoms

#### Description of survivors and prevalence of post-viral symptoms

Of the 138 people included in the post-viral symptoms analysis, the median number of days from ETC discharge to first survivor clinic visit was 109 (IQR 91–210) (Table 2). The majority (64%) of people attended the clinic on two occasions, with the remainder either attending only once (30%) or three times (6%). (S2 Table). Any ocular symptom (100/137 of people who had any symptom data recorded, 73%), any musculoskeletal symptom (107/137, 78%) and headache (63/80, 79%) were the most commonly self-reported problems, with photophobia the most commonly reported specific ocular symptom (46%) and joint pain the most commonly reported musculoskeletal symptom (62%). Excess hunger (99/137, 72%), hair loss (61/137, 45%), fever (55/137, 40%), and dry mouth (52/137, 38%) within the previous seven days were also commonly reported. Hearing loss or tinnitus was reported by 30/137 people (22%).

**Table 2:** Demographic information and self-reported symptoms in 138 attendees of the Kerry Town Ebola survivors clinic.

	N*	n^†^	%
**Total**	138	138	100
**Median age (IQR) years**	138	21 (14–30)	-
**Female**	138	78	57
**Median days from ETC discharge to first clinic visit (IQR)**	138	109	91–120
**Referred to specialist clinic**^‡^	138	68	49
**Self reported symptoms at survivor clinic**^§^			
Fever	137	55	40
**Any ocular symptom**^¶^	**137**	**100**	**73**
Ocular pain	136	55	40
Photophobia	136	63	46
Hyperlacrimation	136	50	37
Loss of vision	136	38	28
Foreign body sensation in the eye	134	47	35
Red eye	136	43	32
**Any musculoskeletal symptom**^¶^	**137**	**107**	**78**
Joint pain	137	85	62
Back pain	137	59	43
Muscle pain	123	49	40
Movement problems	135	31	23
Jaw pain	137	30	22
Chest pain	137	45	33
Parotid pain	135	24	18
Pain with chewing	137	32	24
Hair loss	137	61	45
Peripheral oedema	137	10	7
Headache^#^	80	63	79
Memory loss/disorientation	134	31	23
Hearing loss or tinnitus	137	30	22
Excess hunger/voracious appetite	137	99	72
Abnormal/foul taste or change in taste	136	30	22
Dry mouth	137	52	38
Genital problems	135	24	18
Amenorrhea	77	14	18
Testicular pain	60	3	5
Testicular oedema	60	2	3

*: Total number of people with data for the specific variable.

^†^: Number of people who had the variable in question (e.g. for symptoms, n = number who reported having had the symptom in the previous 7 days).

^‡^: Clinic attendee symptoms required referral to specialist clinic based on symptoms they presented with at the survivor clinic

^§^: Clinic attendee questioned to see if they had had any of the listed symptoms within the previous 7 days

^¶^: Post-viral symptom outcomes selected for further analysis. The 6 eye-related symptoms were combined into the composite outcome “Any ocular symptom” (meaning that a person had at least one of the following symptoms: ocular pain, photophobia, hyperlacrimation, loss of vision, foreign body sensation in the eye, or red eye) while the 5 musculoskeletal-related symptoms were combined into the composite outcome “Any musculoskeletal symptom” (meaning that a person had at least one of the following symptoms: joint pain, back pain, muscle pain, movement problems, jaw pain)

^#^: Data only available for headache for 80 people due to early version of data collection forms not including headache as a symptom.

#### Analysis of risk factors for ocular or musculoskeletal post-viral symptoms

Results of multivariable logistic regression for the ocular and musculoskeletal post-viral outcomes are shown in Table 3. Age was a predictor of musculoskeletal symptoms (with younger age being protective). Among signs and symptoms during acute-phase admission, there was a suggestion that hiccups was associated with a higher risk of ocular symptoms, although confidence intervals spanned the null value. There was weak evidence that female gender predictor ocular symptoms. We did not find evidence that viral load on admission predicted the occurrence of either ocular (1.17, 95% CI 0.35–3.97, comparing low to high CT value) or musculoskeletal (1.07, 95% CI 0.28–4.08) post-viral outcomes in our analysis, although wide confidence intervals meant that we were unable to rule out an increased effect (of up to approximately 4 times the odds) for both outcomes.

**Table 3:** EVD acute-phase predictors for ocular or musculoskeletal post-viral symptoms amongst people admitted to the Kerry Town ETC who survived and attended at least 1 Kerry Town EVD survivor clinic.

	N^†^ (%)	Any ocular symptom*			Any musculoskeletal symptom*		
		n^†^ (%)	Crude OR^‡^ (95% CI)	MV^§^OR (95% CI)	n (%)	Crude OR (95% CI)	MV OR (95% CI)
**Total**	137 (100)	100 (73)	-	-	107 (78)	-	-
**Days admitted**^¶^ (n = 135)^#^:							
median (IQR)	9 (6–14)	9 (6–14)	0.99 (0.93–1.06)	-	10 (5–14)	1.06 (0.98–1.15)	1.09 (0.99–1.19)
**Days since discharge:**							
median (IQR)	109 (91–120)	108 (91–121)	1.00 (0.99–1.01)	-	110 (86–123)	1.00 (0.99–1.01)	-
**Female gender**	77 (56)	60 (78)	1.76 (0.83–3.77)	2.31 (0.98–5.43)	61 (79)	1.16 (0.51–2.62)	-
**Age:**							
<5	9 (7)	6 (67)	1.00 (0.17–5.88)	-	4 (44)	0.09 (0.02–0.58)	0.09 (0.01–0.63)
5–14	32 (23)	25 (78)	1.20 (0.42–3.41)	-	21 (66)	0.45 (0.16–1.30)	0.29 (0.08–1.04)
15–24	40 (29)	28 (70)	1	-	33 (82)	1	1
25–34	30 (22)	22 (73)	1.14 (0.38–3.40)	-	25 (83)	1.35 (0.36–5.02)	0.78 (0.17–3.51)
35–44	15 (11)	10 (67)	0.73 (0.22–2.43)	-	13 (87)	1.76 (0.33–9.32)	1.32 (0.22–7.95)
45+	11 (8)	9 (82)	2.00 (0.38–10.51)	-	11 (100)	-^¶¶^	-
RT-PCR** (n = 110)							-
High	44 (40)	33 (75)	1	1	34 (77)	1	1
Med	36 (33)	23 (64)	0.64 (0.25–1,67)	0.68 (0.24–1.89)	27 (75)	0.90 (0.33–2.47)	0.98 (0.28–3.39)
Low	30 (27)	22 (73)	0.92(0.31–2.69)	1.17 (0.35–3.97)	24 (80)	1.24 (0.41–3.75)	1.07 (0.28–4.08)
**Fever**^††^ (n = 126)	112 (89)	83 (74)	0.92 (0.24–3.46)	-	86 (77)	0.47 (0.10–2.28)	-
**Fatigue/weakness** (n = 126)	109 (87)	83 (76)	1.80 (0.60–5.42)	-	84 (77)	0.75 (0.20–2.77)	-
**Vomit/nausea** (n = 126)	83 (66)	60 (72)	0.64 (0.27–1.52)	0.40 (0.15–1.10)	64 (77)	0.88 (0.36–2.14)	-
**Diarrhoea** (n = 126)	75 (60)	54 (72)	0.66 (0.29–1.50)	-	61 (81)	1.64 (0.70–3.85)	2.37 (0.86–6.51)
**Conjunctivitis/Red eye**^‡‡^ (n = 126)	57 (45)	44 (77)	1.44 (0.65–3.21)	-	48 (84)	2.16 (0.90–5.21)	2.44 (0.88–6.75)
**Muscle/joint pain** (n = 126)	85 (67)	66 (78)	1.86 (0.83–4.16)	2.04 (0.78–5.28)	66 (78)	0.98 (0.40–2.41)	-
**Headache** (n = 126)	85 (67)	63 (74)	1.03 (0.43–2.46)	-	64 (75)	0.65 (0.26–1.65)	-
**Diff breathing** (n = 126)	23 (18)	18 (78)	1.40 (0.48–4.09)	-	16 (70)	0.58 (0.21–1.60)	0.25 (0.06–0.97)
**Skin rash** (n = 126)	5 (4)	5 (100)	-^¶¶^	-	5 (100)	-^¶¶^	-
**Hiccups** (n = 126)	20 (16)	18 (90)	3.59 (0.78–16.45)	4.73 (0.90–24.73)	18 (90)	2.81 (0.60–13.04)	6.25 (0.80–48.89)
**Bleeding**^§§^(n = 126)	11 (9)	8 (73)	0.90 (0.22–3.66)	-	10 (91)	2.71 (0.33–22.12)	-
**Confusion**^‡‡^ (n = 126)	1 (1)	1 (100)	-^¶¶^	-	1(100)	-^¶¶^	-

*: Self-reported. See Table 2 for list of symptoms.

^†^: N(%) = total number of people with potential predictor characteristic or symptom (column %), n(%) = number of people for each potential predictor who had the outcome (row %)

^‡^: Odds Ratio (95% confidence interval). Multiple imputation (MI) used to account for missing data. MI model included all variables in this table except skin rash, confusion and the outcome.

^§^: MV = Multivariable regression model. Model included all variables with results in this column (with variables selected for inclusion from an initial model adjusted for all variables except skin rash and confusion, using a backward stepwise approach, removing variables with p>0.2). Days admitted, age and time since discharge were included as continuous variables (categorical results presented to aid interpretation of results).

^¶^: Days admitted = length of stay at ETC receiving clinical care during Ebola acute-phase of infection.

^#^: The figures in parentheses indicate the total number of individuals for whom this data was available from acute phase (ETC) records. Missing values were imputed using multiple imputation (see note 1). See S4 Table for a comparison of analysing the imputed data versus complete records only.

**: First recorded EBOV RT-PCR cycle threshold at ETC (inverse indicator of viral load), categorised into tertiles of the distribution of the variable (Low: <18.6 cycles, medium: 18.6-<22.5 cycles, high: ≥22.5 cycles).

^††^: All symptoms column 1: recorded by clinical staff on presentation at the Ebola Treatment Centre.

^‡‡^: Data only captured on presentation (not available for capture on standardised forms as an inpatient).

^§§^: Unexplained bleeding.

^¶¶^: Could not be estimated due to low numbers.

### Missing data and sensitivity analysis

Our complete-records sensitivity analyses showed minimal differences from the analysis of our imputed datasets (S3 and S4 Tables). Including RT-PCR CT value as a continuous variable in our model or not including it had a negligible impact on results. The results of repeating the analyses using the lowest RT-PCR CT value during ETC admission (rather than the first) and including the occurrence of symptoms at any time during ETC admission (rather than just on presentation) are included in the S5 and S6 Tables. For all analyses, results were similar with the exception of unexplained bleeding and mortality: the presence of bleeding at any time during ETC admission was associated with a three-fold increase in the odds of mortality 3.08 (1.06–8.90).

## Discussion

In this study, we examined both predictors of mortality and predictors of sequelae from EVD within the same large cohort of patients cared for in a single ETC during both their acute illness and their recovery. Exploration of this single patient population may remove confounding due to systematic differences among treatment centres and cohorts. While high viral load on admission predicted mortality, it did not predict post-viral symptoms. Results from our post hoc analysis suggest that there may be CT value thresholds below and above which death or survival are highly likely (within comparable care settings). People who presented at the ETC in a confused state had a higher odds of death, as did those who suffered from unexplained bleeding at any time during ETC stay (as opposed to upon presentation at ETC). Apart from age (which was a predictor of both mortality and musculoskeletal symptoms), there were no factors that predicted both mortality and the occurrence of post-viral symptoms. There was a suggestion that hiccups predicted post-viral ocular symptoms, and weak evidence for an association between ocular symptoms and gender.

### Comparison with previous studies

#### Predictors of mortality

Our finding that a low CT value (i.e. high viral load) on admission predicts mortality is consistent with other studies of the association[4,19,28–32]. A number of previous studies have shown older and younger age to predict mortality [3,15,17,29,30,32]–in our study we found a strong association with older age, but were underpowered to detect an effect in those under the age of 15 (although notably could not rule out an association as large as 11.64 in the under 5 year old age group). In common with our study, groups who recorded bleeding on triage tended not to find an association with mortality[29,30] while those who recorded bleeding at any time during hospitalisation did find an association[32–34]. This is probably because bleeding indicates severe disease, and people already bleeding in the community are less likely to survive to present at an ETC. Our finding that confusion is a strong predictor for mortality is consistent with other studies (30,35). Our finding that people admitted in later months were more likely to survive could reflect earlier detection of cases in the community (therefore people receive medical care at the ETC earlier in the course of the disease as time goes on), or improving care generally at the ETC over time. The result is also consistent with other studies that have suggested this could be survival bias: patients presenting earlier are likely to be local to the ETC and present early in the course of the disease, whereas those presenting later are from further away, meaning early deaths occur in the community or at holding centres, rather than at the ETC [35,36].

In contrast to previous studies[17,33,34,37], we did not find that diarrhoea predicted death. This discrepancy could be explained by: (i) difference in ages of the cohorts under study (i.e. children are more susceptible to the effects of diarrhoea)[17], (ii) presentation of only unadjusted results in other studies[34], or (iii) potential differences in patient management in other settings compared to Kerry Town where use of IV fluids was part of standard care.

#### Symptoms in survivors and their predictors

In common with our study, a number of studies found that musculoskeletal symptoms were amongst the most frequent problems experienced by survivors [8–10,14]. Ocular symptoms in survivors are also commonly reported elsewhere[8–10,14].

In common with several previous studies[9,10], we did not observe an association between viral load and either ocular or musculoskeletal sequelae. One study from Port Loko, Sierra Leone, has reported a dose-response association between viral load and ocular outcomes[8], though in that study ocular outcomes were ascertained during ETC admission as well as post-discharge, and included assessment of visual acuity and slit-lamp examinations.

Two previous studies [8,10] found that women had an increased risk of ocular symptoms, though relative risks were imprecise. To our knowledge there are no other studies that have studied hiccups as a potential predictor of post-viral symptoms.

### Implications and further work

One hypothesis for the underlying cause of EVD post-viral symptoms is persistence of the virus within immune privileged sites[38], which is more likely in patients with high viral load and severe, prolonged disease[39,40]. An alternative (but not necessarily exclusive) theory is based upon the observation that EBOV infection results in substantial immune activation[40], and that it is this that causes the observed post-viral symptoms[8].

Our findings are more consistent with the latter theory: we found that viral load on admission was not predictive of post-viral symptoms. The suggestion of greater risk of ocular sequelae among women is in line with gender imbalances in a number of immuno-inflammatory conditions (including chronic fatigue syndrome/myalgic encephalopathy, rheumatoid arthritis, and multiple sclerosis)[41,42], and emerging understanding of the impact of sex on immune function during viral infection[43].

The similarity of some post-EVD sequelae to the symptoms of chronic fatigue syndrome has been noted previously, and suggestions have been made that Ebola survivors could be managed by approaches similar to those used for chronic fatigue syndrome or alternatively treated prophylactically with disease-modifying anti-rheumatic drugs (e.g. sulfasazine)[7,44–46]. Furthermore, insatiable hunger, weight loss, palpitations and fever are symptoms of hyperthyroidism, while hair loss, memory loss, low mood, arthralgia and amenorrhoea are symptoms of hypothyroidism (with eye problems symptomatic of both conditions). Since animal models of EBOV infection have demonstrated thyroiditis, further evaluation of thyroid function in survivors is warranted[47].

Our finding of a potential role of hiccups in predicting post-viral ocular symptoms is somewhat difficult to explain. Hiccups can be caused by acute renal failure, central nervous system irritation and paralytic ileus, all of which have been described in acute EVD and which certainly could be associated with a high level of systemic inflammation[19,48,49]. The observation that higher viral load on admission was not associated with survivor symptoms but hiccups were may support the hypothesis that survivor symptoms are an immune-mediated phenomenon rather than a consequence of viral persistence. We should also note that the confidence intervals were wide and multiple comparisons were made, so this could be a chance finding.

### Limitations

Our study has a number of important limitations. Wide confidence intervals for many of the associations we explored may be due to low power (i.e. cohort size), and should thus be interpreted with caution. All of the information for patients with EVD was captured under extreme conditions in an emergency setting and was retrospectively obtained from routinely collected records, meaning that data quality may be an issue.

We chose to base our main analysis on ETC risk factor data obtained upon presentation at ETC in order to minimise unobserved confounding related to factors such as the impact of treatment over time. This does mean that our analysis is susceptible to a degree of measurement error (e.g. some people not displaying a specific symptom at presentation may go on to exhibit it). However, apart from the bleeding finding discussed above, our sensitivity analysis using data obtained anytime during ETC admission did not lead to alternative conclusions.

We did not seek to characterise observed sequelae by analysing laboratory test results, as these were not available for the majority of our cohort. It is difficult to be certain that all post-viral outcomes were EVD, as we lacked a local healthy population control group. While the prevalences found were similar to other studies of EVD survivors, those also lacked control groups. Eliciting history of symptoms during survivor clinics may likewise suffer from response biases related to poor communication of biomedical concepts or perceived incentives to over- or under-report. Our estimates were similar to other studies that did include results from diagnostics, however.

It was not possible in our study to assess the extent to which survivor symptoms improved or worsened over time, and the limited number of survivor follow-up visits and relatively short follow-up time mean that we may have missed some survivor outcomes. We also had to make a pragmatic decision about what type of survivor symptom outcomes to study, could not study all available recorded post-viral symptoms as outcomes (for which there may be different predictors), and did not have access to results of specialised consultations for patients who were referred elsewhere from the survivor clinic (meaning we would not know if a patient went on to be diagnosed with severe sequelae such as e.g. severe neurological complications, depression, arthritis, or uveitis). Furthermore, in this study we have focused only on the clinical symptoms of survivors. We acknowledge that the psychosocial aspects of recovery are equally important to the well-being of those surviving the disease.

Finally, our patient population may not be representative of the entire EVD epidemic, though this is counterbalanced by being able to follow a single cohort from admission at ETC until reporting of post-viral symptoms.

### Conclusions

In this study we were able to examine risk factors for both mortality and sequelae from EVD in a single large cohort. Although viral load in the acute phase of EVD (upon ETC admission) predicted mortality, surprisingly we did not find it to be an important predictor of ocular or musculoskeletal symptoms in survivors. In contrast, female gender was predictive of ocular sequelae, and there was weak evidence that hiccups was also predictive These findings may add epidemiological support to the hypothesis that post-viral symptoms have an immune-mediated aspect and may not only be a consequence of high viral load and disease severity.

As evidence accumulates from different study sites, we believe that a systematic review and meta-analysis of post-viral symptoms and their predictors would be worthwhile, as would pooled analysis: the latter, in particular, would resolve possible issues with low study power. Such summary analyses, however, would benefit from standardised case definitions of health problems being studied and time criteria, e.g. for what constitutes the acute and post-viral phases. It will also be essential to include a population control group as many of the symptoms may be common in the general population as well.

## Supporting information

S1 Table STROBE statement—Checklist of items that should be included in reports of *cohort studies*. (DOC)

S2 Table Number and frequency of visits to the survivor clinic (N = 138). (DOCX)

S3 Table Predictors of mortality analysis—Comparison of multiple imputation analysis with a complete records analysis for variables with missing data (N = 263). (DOCX)

S4 Table Predictors of postviral symptoms analysis—Comparison of multiple imputation analysis with a complete records analysis for variables with missing data (N = 137). (DOCX)

S5 Table Predictors for mortality amongst all EVD-positive people admitted to Kerry Town ETC, using data on symptoms collected at any time during ETC stay and the lowest recorded viral load during ETC stay. (DOCX)

S6 Table EVD acute-phase predictors for ocular or musculoskeletal post-viral symptoms amongst people admitted to the Kerry Town ETC who survived and attended at least 1 Kerry Town EVD survivor clinic, using data on symptoms collected at any time during ETC stay and the lowest recorded viral load during ETC stay. (DOCX)

S1 Text Supplementary information for materials and methods. (DOCX)

S2 Text References for supporting information. (DOCX)

S1 Data Minimal underlying data. (XLS)
