# Supplementary material for: Surviving Ebola: A historical cohort study of Ebola mortality and survival in Sierra Leone 2014-2015
Source: PLoS One. 2018 Dec 27;13(12):e0209655. doi: 10.1371/journal.pone.0209655 (PMC6307710; doi:10.1371/journal.pone.0209655)
Supplement: S2 Table — (DOCX) [file pone.0209655.s002.docx]

S2 Table: Number and frequency of visits to the survivor clinic (N=138)

|  |  |  |
| --- | --- | --- |
|  |  |  |
|  | **n (%)** |  |
| **Number of visits** |  |  |
| 1 | 42 (30) |  |
| 2 | 88 (64) |  |
| 3 | 8 (6) |  |
|  |  |  |
|  | **Median (IQR)** |  |
| Amongst all attendees (n=138) |  |  |
| **Number of days between clinic opening and first visit** | 14 (8 - 21) |  |
|  |  |  |
| Amongst those who attended twice (n=88) |  |  |
| **Number of days between first and second visits** | 36 (28 – 48) |  |
| **Number of days between last visit and date clinic closed** | 40 (32-49) |  |
|  |  |  |
| Amongst those who attended three times (n=8) |  |  |
| **Number of days between first and second visits** | 32 (22 – 35) |  |
| **Number of days between second and third visits** | 16 (14 - 19) |  |
| **Number of days between last visit and date clinic closed** | 24 (19 - 32) |  |
|  |  |  |
