# Supplementary material for: Surviving Ebola: A historical cohort study of Ebola mortality and survival in Sierra Leone 2014-2015
Source: PLoS One. 2018 Dec 27;13(12):e0209655. doi: 10.1371/journal.pone.0209655 (PMC6307710; doi:10.1371/journal.pone.0209655)
Supplement: S3 Table — (DOCX) [file pone.0209655.s003.docx]

S3 Table: Predictors of mortality analysis – comparison of multiple imputation analysis with a complete records analysis for variables with missing data (N=263)

|  |  | Crude Odds Ratio (95% confidence interval) | |
| --- | --- | --- | --- |
|  |  | **Complete records^1^** | **Multiple imputation^2^** |
| **RT-PCR cycle** | High | 1 | 1 |
| **Threshold** (n=214) | Med | 3.71 (1.62 – 8.50) | 3.71 (1.61- 8.54) |
|  | Low | 39.44 (15.35 – 101.36) | 32.85 (12.61-85.60) |
| **Fever** (n=242) | No | 1 | 1 |
|  | Yes | 0.80 (0.37 - 1.71) | 0.83 (0.39-1.76) |
| **Fatigue/weakness** (n=242) | No | 1 | 1 |
|  | Yes | 1.20 (0.54 - 2.66) | 1.18 (0.54-2.58) |
| **Vomiting/nausea** (n=242) | No | 1 | 1 |
|  | Yes | 0.94 (0.55 - 1.62) | 0.93 (0.54-1.60) |
| **Diarrhoea** (n=242) | No | 1 | 1 |
|  | Yes | 1.28 (0.75 - 2.17) | 1.24 (0.72-2.16) |
| **Conjunctivitis/Red eye** (n=242) | No | 1 | 1 |
|  | Yes | 1.48 (0.88 - 2.48) | 1.46 (0.86-2.49) |
| **Muscle/joint pain** (n=242) | No | 1 | 1 |
|  | Yes | 0.86 (0.51 - 1.48) | 0.86 (0.51-1.46) |
| **Headache** (n=242) | No | 1 | 1 |
|  | Yes | 0.60 (0.35-1.01) | 0.60 (0.35-1.02) |
| **Difficulty breathing** (n=242) | No | 1 | 1 |
|  | Yes | 0.82 (0.42 - 1.59) | 0.81 (0.42-1.57) |
| **Skin rash** (n=242) | No | 1 | 1 |
|  | Yes | 0.93 (0.25 - 3.38) | 0.93 (0.26-3.32) |
| **Hiccups** (n=242) | No | 1 | 1 |
|  | Yes | 1.24 (0.62 - 2.47) | 1.29 (0.66-2.53) |
| **Unexplained bleeding** (n=242) | No | 1 | 1 |
|  | Yes | 1.90 (0.83 - 4.38) | 1.94 (0.85-4.44) |
| **Confusion** (n=242) | No | 1 | 1 |
|  | Yes | 11.18 (2.48-50.41) | 11.29 (2.47-51.54) |
| **Note 1:** Complete records – only individuals with complete records for the variable in question were analysed. **Note 2:** Multiple imputation (MI) used to account for missing data, with variables included in the MI model as per Table 1 (Note 2). | | | |
