# Supplementary material for: Surviving Ebola: A historical cohort study of Ebola mortality and survival in Sierra Leone 2014-2015
Source: PLoS One. 2018 Dec 27;13(12):e0209655. doi: 10.1371/journal.pone.0209655 (PMC6307710; doi:10.1371/journal.pone.0209655)
Supplement: S4 Table — (DOCX) [file pone.0209655.s004.docx]

**S4 Table: Predictors of postviral symptoms analysis –**

**comparison of multiple imputation analysis with a complete records analysis for variables with missing data (N=137)**

|  |  | **Any ocular symptom**Crude OR (95% CI) | |  | **Any musculoskeletal symptom**Crude OR (95% CI) | | |  |
| --- | --- | --- | --- | --- | --- | --- | --- | --- |
|  |  | Complete records^1^ | Multiple imputation^2^ |  | Complete records | Multiple imputation |  |  |
| **Days ill** Median (IQR) (n=135) |  | 1.19 (0.55-2.56) | 0.99 (0.93-1.06) |  | 1.06 (0.98-1.15) | 1.09 (0.99-1.19) |  |  |
| **RT-PCR** (n=110) |  |  |  |  |  |  |  |  |
| High |  | 1 | 1 |  | 1 | 1 |  |  |
| Med |  | 0.59 (0.23 - 1.54) | 0.64 (0.25-1,67) |  | 0.88 (0.31 – 2.48) | 0.90 (0.33-2.47) |  |  |
| Low |  | 0.92 (0.32 - 2.64) | 0.92 (0.31-2.69) |  | 1.18 (0.38 – 3.68) | 1.24 (0.41-3.75) |  |  |
| **Fever** (n=126) |  | 0.78 (0.20 - 3.00) | 0.92 (0.24-3.46) |  | 0.55 (0.12 - 2.62) | 0.47 (0.10-2.28) |  |  |
| **Fatigue/weakness** (n=126) |  | 1.74 (0.59 - 5.17) | 1.80 (0.60-5.42) |  | 0.72 (0.19 - 2.71) | 0.75 (0.20-2.77) |  |  |
| **Vomiting/nausea** (n=126) |  | 0.69 (0.29 - 1.66) | 0.64 (0.27-1.52) |  | 0.89 (0.36 - 2.18) | 0.88 (0.36-2.14) |  |  |
| **Diarrhoea** (n=126) |  | 0.71 (0.31 - 1.63) | 0.66 (0.29-1.50) |  | 1.65 (0.71 - 3.84) | 1.64 (0.70-3.85) |  |  |
| **Conjunctivitis** (n=126) |  | 1.38 (0.62 - 3.10) | 1.44 (0.65-3.21) |  | 2.18 (0.90 - 5.26) | 2.16 (0.90-5.21) |  |  |
| **Muscle/joint pain** (n=126) |  | 1.61 (0.70 - 3.71) | 1.86 (0.83-4.16) |  | 0.98 (0.40 - 2.40) | 0.98 (0.40-2.41) |  |  |
| **Headache** (n=126) |  | 0.92 (0.39-2.19) | 1.03 (0.43-2.46) |  | 0.63 (0.24-1.62) | 0.65 (0.26-1.65) |  |  |
| **Difficulty breathing** (n=126) |  | 1.28 (0.43 - 3.78) | 1.40 (0.48-4.09) |  | 0.59 (0.21 - 1.61) | 0.58 (0.21-1.60) |  |  |
| **Hiccups** (n=126) |  | 3.55 (0.78 - 16.26) | 3.59 (0.78-16.45) |  | 2.93 (0.64 - 13.46) | 2.81 (0.60-13.04) |  |  |
| **Bleeding** (n=126) |  | 0.90 (0.22 - 3.62) | 0.90 (0.22-3.66) |  | 3.07 (0.38 - 25.07) | 2.71 (0.33-22.12) |  |  |
| **Note 1:** Complete records – only individuals with complete records for the variable in question were analysed. **Note 2:** Multiple imputation (MI) used to account for missing data, with variables included in the MI model as per Table 2 (Note 2). | | | | | | | | |
