# Supplementary material for: Surviving Ebola: A historical cohort study of Ebola mortality and survival in Sierra Leone 2014-2015
Source: PLoS One. 2018 Dec 27;13(12):e0209655. doi: 10.1371/journal.pone.0209655 (PMC6307710; doi:10.1371/journal.pone.0209655)
Supplement: S5 Table — (DOCX) [file pone.0209655.s005.docx]

**S5 Table: Predictors for mortality amongst all EVD-positive people admitted to Kerry Town ETC, using data on symptoms collected at any time during ETC stay and the lowest recorded viral load during ETC stay.**

|  |  | **All**  n (%) | **Recovered**  n (%) | **Died** | **Crude OR^1^**  **(95% CI^2^)** | **Multivariable^3^**  **OR (95% CI)** |  |
| --- | --- | --- | --- | --- | --- | --- | --- |
| **Total^4^** |  | 263 (100) | 152 (58) | 111 (42) | - | - |  |
| **Gender** | Male | 115 (44) | 60 (52) | 55 (48) | 1 | - |  |
|  | Female | 148 (56) | 92 (62) | 56 (38) | 0.66 (0.41 – 1.09) | - |  |
| **Age in years** | <5 | 21 (8) | 10 (48) | 11 (52) | 2.82 (1.19 – 7.00) | 2.59 (0.42-15.80) |  |
|  | 5-14 | 48 (18) | 35 (73) | 13 (27) | 0.95 (0.40 – 2.25) | 1.02 (0.27-3.85) |  |
|  | 15-24 | 57 (22) | 41 (72) | 16 (28) | 1 | 1 |  |
|  | 25-34 | 71 (27) | 34 (48) | 37 (52) | 2.79 (1.33 – 5.86) | 6.92 (1.98-24.19) |  |
|  | 35-44 | 34 (13) | 16 (47) | 18 (53) | 2.88 (1.19 – 7.00) | 9.45 (2.08-43.02) |  |
|  | 45+ | 32 (12) | 16 (50) | 16 (50) | 2.56 (1.04 – 6.32) | 18.82 (3.62-97.73) |  |
| **Date of** | Nov 14 | 42 (16) | 14 (33) | 28 (67) | 1 | 1 |  |
| **Admission** | Dec 14 | 160 (61) | 100 (63) | 60 (38) | 0.30 (0.15 – 0.61) | 0.18 (0.05-0.61) |  |
|  | Jan 15 | 34 (13) | 22 (65) | 12 (35) | 0.27 (0.11 – 0.71) | 0.15 (0.03-0.72) |  |
|  | Feb/Mar 15 | 27 (10) | 16 (59) | 11 (41) | 0.34 (0.13 – 0.93) | 0.37 (0.07-2.05) |  |
| **RT-PCR cycle** | High | 72 (34) | 63 (88) | 9 (12) | 1 | 1 |  |
| **threshold^5^** (n=214)^6^ | Med | 71 (33) | 49 (69) | 22 (31) | 3.30 (1.44-7.56) | 5.91 (1.91-18.27) |  |
|  | Low | 71 (33) | 10 (14) | 61 (86) | 37.28 (14.81-93.85) | 131.54 (30.50-567.21) |  |
| **Fever^7^** (n=250) | No | 26 (10) | 16 (62) | 10 (38) | 1 | - |  |
|  | Yes | 224 (90) | 131 (58) | 93 (42) | 1.13 (0.49-2.58) | - |  |
| **Fatigue/weakness** (n=251) | No | 16 (6) | 10 (63) | 6 (38) | 1 | - |  |
|  | Yes | 235 (94) | 138 (59) | 97 (41) | 1.15 (0.41-3.26) | - |  |
| **Vomiting/nausea** (n=250) | No | 54 (22) | 33 (61) | 21 (39) | 1 | - |  |
|  | Yes | 196 (78) | 114 (58) | 82 (42) | 1.13 (0.62-2.07) | - |  |
| **Diarrhoea** (n=250) | No | 45 (18) | 29 (64) | 16 (36) | 1 | - |  |
|  | Yes | 205 (82) | 118 (58) | 87 (42) | 1.34 (0.69-2.61) | - |  |
| **Conjunctivitis/red eye** (n=242) | No | 145 (60) | 90 (62) | 55 (38) | 1 | - |  |
|  | Yes | 97 (40) | 51 (53) | 46 (47) | 1.51 (0.90-2.54) | - |  |
| **Muscle/joint pain** (n=251) | No | 52 (21) | 26 (50) | 26 (50) | 1 | 1 |  |
|  | Yes | 199 (79) | 122 (61) | 77 (39) | 0.62 (0.34-1.13) | 0.39 (0.16 – 0.98) |  |
| **Headache** (n=250) | No | 72 (29) | 32 (44) | 40 (56) | 1 | 1 |  |
|  | Yes | 178 (71) | 115 (65) | 63 (35) | 0.43 (0.25-0.75) | 0.16 (0.06-0.44) |  |
| **Difficulty breathing** (n=250) | No | 177 (71) | 107 (60) | 70 (40) | 1 | - |  |
|  | Yes | 73 (29) | 40 (55) | 33 (45) | 1.26 (0.72 – 2.18) | - |  |
| **Skin rash** (n=250) | No | 240 (96) | 141 (59) | 99 (41) | 1 | - |  |
|  | Yes | 10 (4) | 6 (60) | 4 (40) | 0.98 (0.27 – 3.60) | - |  |
| **Hiccups** (n=250) | No | 201 (80) | 122 (61) | 79 (39) | 1 | - |  |
|  | Yes | 49 (20) | 25 (51) | 24 (49) | 1.46 (0.77 – 2.73) | - |  |
| **Unexplained bleeding** (n=250) | No | 202 (81) | 130 (64) | 72 (36) | 1 | 1 |  |
|  | Yes | 48 (19) | 17 (35) | 31 (65) | 3.27 (1.6 8 – 6.34) | 3.08 (1.06-8.90) |  |
| **Confusion** (n=250) | No | 234 (94) | 145 (62) | 89 (38) | 1 | 1 |  |
|  | Yes | 16 (6) | 2 (13) | 14 (88) | 11.23 (2.35-53.61) | 11.48 (1.40-94.39) |  |
| **Note 1:** Odds ratio. Multiple imputation (MI) used to account for missing data for all variables with missing data. MI model included all variables in this table and the outcome status. **Note 2:** Confidence interval **Note 3:** An initial multivariable regression model was prepared that included all variables in this table. The final model presented here was obtained by removing variables from the initial fully-adjusted model in a backwards stepwise fashion, keeping only those variables with p≤0.2. Age was included as a continuous variable (multivariable-adjusted categorical results presented to aid interpretation of results). **Note 4:** Total = total number of EBOV-positive people admitted to Kerry Town ETC **Note 5:** Lowest recorded RT-PCR cycle threshold value at ETC (inverse indicator of viral load), categorised into tertiles of the distribution of the variable (Low : <18.6 cycles, medium: 18.6-<22.5 cycles, high: ≥22.5 cycles). **Note 6:** The figures in brackets indicate the total number of individuals with any data recorded for that variable. Missing values for all variables with missing data were imputed using multiple imputation (see note 1). **Note 7:** All symptoms in this table: recorded anytime during ETC stay. | | | | | | | |
