# Supplementary material for: Surviving Ebola: A historical cohort study of Ebola mortality and survival in Sierra Leone 2014-2015
Source: PLoS One. 2018 Dec 27;13(12):e0209655. doi: 10.1371/journal.pone.0209655 (PMC6307710; doi:10.1371/journal.pone.0209655)
Supplement: S6 Table — (DOCX) [file pone.0209655.s006.docx]

**S6 Table: EVD acute-phase predictors for ocular or musculoskeletal post-viral symptoms amongst people admitted to the Kerry Town ETC who survived and attended at least 1 Kerry Town EVD survivor clinic, using data on symptoms collected at any time during ETC stay and the lowest recorded viral load during ETC stay**

|  |  | Any ocular symptom^1^ | | | |  | Any musculoskeletal symptom^1^ | | | | |  |
| --- | --- | --- | --- | --- | --- | --- | --- | --- | --- | --- | --- | --- |
|  |  | No | Yes | Crude OR (95% CI)^2^ | MV^3^ OR (95% CI) |  | | No | Yes | Crude OR (95% CI) | MV OR (95% CI) | |
|  | n (%) | n (%) | n (%) |  |  |  | | n (%) | n (%) |  |  | |
| **Total^4^** | 137 (100) | 37 (27) | 100 (73) | - | - |  | | 30 (22) | 107 (78) | - | - | |
| **Days ill^5^** (n=135)^6^**:** |  |  |  |  |  |  | |  |  |  |  | |
| median (IQR) | 9 (6-14) | 8 (6-14) | 9 (6-14) | 0.99 (0.93-1.06) | - |  | | 8 (7-9) | 10 (5-14) | 1.06 (0.98-1.15) | 1.09 (1.00-1.21) | |
| **Days since discharge:** |  |  |  |  |  |  | |  |  |  |  | |
| median  (IQR) | 109  (91-120) | 110  (93-119) | 108  (91-121) | 1.00  (0.99-1.01) | - |  | | 107  (102-112) | 110  (86-123) | 1.00 (0.99-1.01) | - | |
| **Female gender** | 77 (56) | 17 (22) | 60 (78) | 1.76 (0.83-3.77) | 1.70 (0.77-3.75) |  | | 16 (21) | 61 (79) | 1.16 (0.51-2.62) | - | |
| **Age:** |  |  |  |  |  |  | |  |  |  |  | |
| <5 | 9 (7) | 3 (33) | 6 (67) | 1.00 (0.17-5.88) | - |  | | 5 (56) | 4 (44) | 0.09 (0.02-0.58) | 0.28 (0.01-0.33) | |
| 5-14 | 32 (23) | 7 (22) | 25 (78) | 1.20 (0.42-3.41) | - |  | | 11 (34) | 21 (66) | 0.45 (0.16-1.30) | 0.29 (0.08-1.01) | |
| 15-24 | 40 (29) | 12 (30) | 28 (70) | 1 | - |  | | 7 (18) | 33 (82) | 1 | 1 | |
| 25-34 | 30 (22) | 8 (27) | 22 (73) | 1.14 (0.38-3.40) | - |  | | 5 (17) | 25 (83) | 1.35 (0.36-5.02) | 1.56 (0.36-6.71) | |
| 35-44 | 15 (11) | 5 (33) | 10 (67) | 0.73 (0.22-2.43) | - |  | | 2 (13) | 13 (87) | 1.76 (0.33-9.32) | 1.60 (0.27-9.33) | |
| 45+ | 11 (8) | 2 (18) | 9 (82) | 2.00 (0.38-10.51) | - |  | | 0 (0) | 11 (100) | -^7^ | - | |
| **RT-PCR^8^** (110) |  |  |  |  |  |  | |  |  |  | - | |
| High | 37 (34) | 9 (24) | 28 (76) | 1 | 1 |  | | 10 (27) | 27 (73) | 1 | 1 | |
| Med | 39 (35) | 14 (36) | 25 (64) | 0.61 (0.23-1.61) | 0.64 (0.24-1.76) |  | | 9 (23) | 30 (77) | 1.20 (0.44-3.28) | 1.83 (0.50-6.70) | |
| Low | 34 (31) | 9 (26) | 25 (74) | 0.82 (0.29-2.37) | 0.81 (0.28-2.39) |  | | 6 (18) | 28 (82) | 1.64 (0.51-5.28) | 2.05 (0.41-10.22) | |
| **Fever^9^** (n=126) | 117 (89) | 31 (26) | 86 (74) | 1.05 (0.31-3.50) | - |  | | 28 (24) | 89 (76) | 0.47 (0.10-2.23) | - | |
| **Fatigue/weakness** (n=126) | 123 (92) | 32 (26) | 91 (74) | 1.24 (0.31-5.03) | - |  | | 29 (24) | 94 (76) | 0.33 (0.04-2.68) | - | |
| **Vomit/nausea** (n=126) | 101 (77) | 29 (29) | 72 (71) | 0.60 (0.23-1.62) | - |  | | 22 (22) | 79 (78) | 1.24 (0.49-3.15) | - | |
| **Diarrhoea** (n=126) | 104 (79) | 29 (28) | 75 (72) | 0.67 (0.25-1.82) | - |  | | 22 (21) | 82 (79) | 1.47 (0.57-3.78) | - | |
| **Conjunctivitis/**  **Red eye^10^** (n=126) | 57 (45) | 13 (23) | 44 (77) | 1.24 (0.54-2.85) | - |  | | 9 (16) | 48 (84) | 1.94 (0.76-4.94) | 2.20 (0.71-6.85) | |
| **Muscle/joint pain** (n=126) | 110 (83) | 29 (26) | 81 (74) | 0.94 (0.34-259.) | - |  | | 26 (24) | 84 (76) | 0.66 (0.21-2.12) | 0.16 (0.03-0.89) | |
| **Headache** (n=126) | 103 (78) | 27 (26) | 76 (74) | 1.11 (0.44-2.78) |  |  | | 24 (23) | 79 (77) | 0.81 (0.30-2.22) |  | |
| **Diff breathing** (n=126) | 32 (24) | 8 (25) | 24 (75) | 1.07 (0.43-2.67) | - |  | | 10 (31) | 22 (69) | 0.55 (0.23-1.35) | 0.39 (0.12-1.25) | |
| **Skin rash** (n=126) | 5 (4) | 0 (0) | 5 (100) | - | - |  | | 4 (17) | 20 (83) | - | - | |
| **Hiccups** (n=126) | 24 (18) | 2 (8) | 22 (92) | 5.00 (1.11-22.54) | 5.13 (1.12-23.51) |  | | 2 (14) | 12 (86) | 1.59 (0.50-5.08) | 3.71 (0.78-17.63) | |
| **Bleeding^11^**(n=126) | 14 (11) | 4 (29) | 10 (71) | 0.88 (0.26-2.98) | - |  | | 28 (24) | 89 (76) | 1.92 (0.41-9.13) | - | |
| **Confusion** (n=126) | 1 (1) | 0 (0) | 1 (100) | - | - |  | | 0 (0) | 1 (100) | - | - | |
| **Note 1:** Self reported. See table 2 for list of specific symptoms. The n(%) column applies to both the Any ocular symptoms columns and the Any musculoskeletal symptom columns (i.e. the same 137 people were included in both analyses). **Note 2:** Odds Ratio (95% confidence interval). Multiple imputation (MI) used to account for missing data for all estimates with missing data. MI model included all variables in this table except for skin rash and the outcome status. **Note 3:** MV=Multivariable regression model. Model included all variables with results in this column (with variables selected for inclusion from an initial model adjusted for all variables except skin rash, using a backward stepwise approach, removing variables with p>0.2). Days ill, age and time since discharge were included as continuous variables (MV-adjusted categorical results age presented to aid interpretation of results). **Note 4:** The totals provided here are the number (out of 138) who had information recorded for the specific post-viral outcome (either ocular symptoms or arthralgia) **Note 5:** Days ill=length of stay at ETC receiving clinical care during Ebola acute-phase of infection. **Note 6:** The figures in brackets indicate the total number of individuals with any data recorded for that variable. **Note 7:** Not possible to estimate due to lack of negative outcomes. **Note 8**: Lowest recorded RT-PCR cycle threshold value while at ETC. Low category <20 cycles, high category≥20 cycles. **Note 9:** All symptoms in first column of this table: recorded by clinical staff at any time during inpatient stay at Ebola Treatment Centre (including on presentation). **Note 10:** Data only captured on presentation (not available for capture on standardised forms as an inpatient). **Note 11:** Unexplained bleeding. | | | | | | | | | | | | |
