# Supplementary material for: Surviving Ebola: A historical cohort study of Ebola mortality and survival in Sierra Leone 2014-2015
Source: PLoS One. 2018 Dec 27;13(12):e0209655. doi: 10.1371/journal.pone.0209655 (PMC6307710; doi:10.1371/journal.pone.0209655)
Supplement: S1 Text — (DOCX) [file pone.0209655.s007.docx]

S1 Text: Supplementary Information for Materials and Methods

Study participants and setting

*Additional details relating to the Kerry Town ETC and the survivor clinic*

The Kerry Town Ebola Treatment Centre (ETC) had suspect wards for patients without an Ebolavirus (EBOV) test result prior to arrival, and treatment wards for those with confirmed Ebola Virus Disease (EVD). Patients arriving at the ETC by ambulance who had already been confirmed as EBOV-positive at other facilities in the area were admitted directly into the confirmed patient wards and a repeat test was taken. Patients with suspected EVD arriving by ambulance, car, taxi or on foot who met the admission criteria at ETC triage were admitted to suspect wards. From November 2014 until early January 2015, the majority of admissions were confirmed positive patients (since the formal capability to accept suspect patients was only introduced in early January). All confirmed and suspected EVD patients received an initial on-site EBOV PCR, with results provided within 24 hours from admission, and those considered to be at stage 2 or above also received broad-spectrum antimicrobial therapy. Suspect patients who tested positive for malaria also received antimalarial drugs. Suspect patients who tested EBOV-positive were transferred to the confirmed patient wards while those testing negative were assessed on a case by case basis. If well enough and/or an alternative diagnosis was made they were treated and discharged. Those with severe illnesses that were not EBOV-related were managed in the suspect area. If required, they were transferred to Connaught Hospital, Free Town, which had the only medical facilities for EBOV-negative patients in the region. EBOV-positive patients admitted to the confirmed patient wards received primarily supportive treatment appropriate to their stage of disease, including oral and intravenous medications, oral rehydration salt solution, and intravenous fluid replacement. EBOV tests were repeated once a patient’s condition improved, and a patient was designated as EBOV-negative and discharged from the ETC if they had two consecutive EBOV-negative test results (taken on two separate occasions) and no EVD symptoms for a period of three days.

At the time of discharge from the ETC, EVD survivors underwent a psychosocial support assessment interview with a trained psychologist using a Krio translator if necessary. Each survivor was provided with a survivor certificate in order to aid integration back into the community. Attempts were subsequently made to contact each discharged survivor at home, in order to request their attendance at a survivor clinic at the Kerry Town ETC, which was open between 2 April and 30 June 2015 (with consultations taking place between 2 April and 24 June). Subsidised transport to the survivor clinic was provided. Patients were screened for acute infection upon presentation at the clinic as described previously(1). Survivors with symptoms that the attending clinician considered required specialist input were referred onto hospitals. A psychosocial assessment was also performed by a trained psychologist (using a translator as needed) at the majority of survivor clinic visits (data not presented here).

Previous studies including some of the same participants

150 of the 263 EBOV+ patients included in this study were included in a previous study of survival within the ETC (2). As well as being much smaller, this study had a greater focus on the involvement of biochemical and haematological parameters than our study, less focus on individual symptoms as predictors of mortality, and did not study survivor symptoms. A number of other studies included all or a subset of the cohort of survivors included in our study.(3–8) While our study focuses on acute-phase predictors of post-viral symptoms, the other studies were investigating viraemia and EBOV secretion (and potential risk for health-care providers), long term mortality, and various aspects of household transmission.

Data collection

Paper medical records were scanned and stored in a password-protected encrypted fileshare. Anonymised data were then entered into a password-protected database (Epi Info version 7.1.4; Centers for Disease Control and Prevention, Atlanta, GA, USA), before importing into Stata (StataCorp. 2015. Stata Statistical Software: Release 14. College Station, TX) for analysis. The data used in this study were limited to information that had been entered into symptom/condition checkboxes or numerical values into standardised forms used for routine data collection at the ETC and survivor clinics – additional notes made by clinicians that were not entered into these specific forms were not available for analysis.

Outcomes and risk factors

Further details on risk factors

Symptoms or signs during acute-phase infection were recorded at the ETC on standardised medical forms by clinicians responsible for patient care. Fever was classified as any documented temperature >37.8C. There was a single medical form entry that covered both fatigue and weakness (subsequently referred to in the article as fatigue/weakness) and also similarly for vomiting or nausea (subsequently referred to as vomiting/nausea), conjunctivitis or red eye (subsequently referred to as conjunctivitis/red eye), muscle or joint pain (subsequently referred to as muscle/joint pain). Whether or not a patient had conjunctivitis/red eye was captured on standardised forms at ETC admission but was not then routinely noted during ETC stay. Patients were considered “confused” if they were disorientated to time, place or person using inappropriate words during communication with the admitting clinician (this was not then routinely noted during ETC stay). Unexplained bleeding meant that bleeding could not be explained by the presence of any cause other than EVD. All other symptoms included in the study were captured on standardised forms at ETC admission and/or at any other time during the ETC stay. A categorical age variable was created by categorizing into those <5 years, then in 10 year age bands up to a 45+ year group. A categorical month of admission group was created, with February and March combined into a single category due to lower numbers in each of these months.

RT-PCR cycle threshold values

The PCR results provided by the KerryTown Ebola Laboratory, and most other laboratories which tested clinical samples during the west-african outbreak, were cycle thresholds. These cannot be converted into RNA copies per ml values retrospectively. In order to present c/ml values for our patient samples, a serial dilution of samples with known Ebola virus c/ml would have to be tested at the same time as each patient sample (i.e. in each ‘run’), all of which would need to be performed in high-level biosafety laboratories. This is outside the scope of this research.

Although EBOV RT-PCR cycle threshold value was a continuous variable, this was handled in the analyses as a categorical variable. We did not present detailed analysis (for example graphs) of this variable as a continuous variable since the test used provides a qualitative result that is difficult to directly equate to quantitative viral load. Lack of between-lab standardisation would make results associated with individual values difficult to interpret, and may allow false conclusions to be drawn. We instead chose to use low, medium and high categorisations, splitting the variable by tertiles of the distribution which ensured sufficient numbers of events within all categories to allow estimation of effects (Low : <18.6 cycles, medium: 18.6-<22.5 cycles, high: ≥22.5 cycles). We considered the inclusion of the variable as a continuous variable in the sensitivity analyses for our multivariable models acceptable because the variable was being included in a model only, and potentially misleading results for specific single units were not being presented.

Postviral symptoms

The standardised list of symptoms for self-report included: fever, joint pain, muscle pain, ocular pain, photophobia, hyperlacrimation/excessive tears, loss of vision, foreign body sensation in the eye, red eye, excessive hunger, hearing loss, genital problems, amenorrhea, testicular pain, testicular oedema, jaw pain, parotid pain, abnormal/foul change in taste, dry mouth, pain with chewing, excessive fatigue, hair loss, edema, memory loss, loss of appetite, abdominal pain, chest pain, back pain, movement problems, and any other reported symptom. The list of symptoms was updated during the study period to include headache, but as this meant that presence or absence of headache was only recorded for 50% of participants, this variable was not included in the analysis.

Statistical analysis

Analysis of risk factors for mortality and postviral symptoms

A relatively large p-value threshold (0.2) was utilized for model preparation based upon guidance related to strategies for predictive modelling in small data sets(9). For any of the continuous variables that were included in the final model, the results of including the specific variables as a categorical variable in the final model were provided in Tables 1, 3 and 4 in order to aid interpretation of results.

Missing data

All available variables were included in the multiple imputation model, and we created 25 imputed datasets, which were combined for analysis. Our missing-at-random assumption was based on a priori reasoning that missing data across all variables would be dependent on other recorded variables, with date of admission considered to be of particular importance because of differences in patient and data handling approaches over time. A complete-records sensitivity analysis was performed, to enable comparison with the results obtained by using multiple imputation (see Results section of this Supporting Information document).
