# Supplementary material for: Surviving Ebola: A historical cohort study of Ebola mortality and survival in Sierra Leone 2014-2015
Source: PLoS One. 2018 Dec 27;13(12):e0209655. doi: 10.1371/journal.pone.0209655 (PMC6307710; doi:10.1371/journal.pone.0209655)
Supplement: S2 Text — (DOCX) [file pone.0209655.s008.docx]

**S2 Text: References for Supporting Information**

1. Green E, Hunt L, Ross JCG, Nissen NM, Curran T, Badhan A, et al. Viraemia and Ebola virus secretion in survivors of Ebola virus disease in Sierra Leone: a cross-sectional cohort study. Lancet Infect Dis. 2016;16(9):1052–6.

2. Hunt L, Gupta-Wright A, Simms V, Tamba F, Knott V, Tamba K, et al. Clinical presentation, biochemical, and haematological parameters and their association with outcome in patients with Ebola virus disease: An observational cohort study. Lancet Infect Dis. 2015 Nov;15(11):1292–9.

3. Ngo ST, Steyn FJ, McCombe PA. Gender differences in autoimmune disease. Front Neuroendocrinol. 2014 Aug 1;35(3):347–69.

4. Bower H, Smout E, Bangura MS, Kamara O, Turay C, Johnson S, et al. Deaths, late deaths, and role of infecting dose in Ebola virus disease in Sierra Leone: retrospective cohort study. BMJ. 2016 May 17;353:i2403.

5. Bower H, Johnson S, Bangura MS, Kamara AJ, Kamara O, Mansaray SH, et al. Effects of Mother’s Illness and Breastfeeding on Risk of Ebola Virus Disease in a Cohort of Very Young Children. Akogun OB, editor. PLoS Negl Trop Dis. 2016 Apr 8;10(4):e0004622.

6. Bower H, Johnson S, Bangura MS, Kamara AJ, Kamara O, Mansaray SH, et al. Exposure-Specific and Age-Specific Attack Rates for Ebola Virus Disease in Ebola-Affected Households, Sierra Leone. Emerg Infect Dis. 2016 Aug;22(8):1403–11.

7. Glynn JR, Bower H, Johnson S, Houlihan CF, Montesano C, Scott JT, et al. Asymptomatic infection and unrecognised Ebola virus disease in Ebola-affected households in Sierra Leone: a cross-sectional study using a new non-invasive assay for antibodies to Ebola virus. Lancet Infect Dis. 2017 Jun 1;17(6):645–53.

8. Glynn JR, Bower H, Johnson S, Turay C, Sesay D, Mansaray SH, et al. Variability in Intrahousehold Transmission of Ebola Virus, and Estimation of the Household Secondary Attack Rate. J Infect Dis. 2018 Jan 4;217(2):232–7.

9. Steyerberg EW, Eijkemans MJC, Harrell FE, Habbema JDF. Prognostic Modeling with Logistic Regression Analysis. Med Decis Mak. 2001 Feb 2;21(1):45–56.
